# Editorial: Environments-pathogens-the gut microbiota and host diseases

**DOI:** 10.3389/fmicb.2023.1357125

**Published:** 2024-01-08

**Authors:** Jinbo Xiong, Zunji Shi

**Affiliations:** ^1^State Key Laboratory for Managing Biotic and Chemical Threats to the Quality and Safety of Agro-products, Ningbo University, Ningbo, China; ^2^Key Laboratory of Marine Biotechnology of Zhejiang Province, School of Marine Sciences, Ningbo University, Ningbo, China; ^3^State Key Laboratory of Herbage Improvement and Grassland Agro-ecosystems, Center for Grassland Microbiome, College of Pastoral Agriculture Science and Technology, Lanzhou University, Lanzhou, China

**Keywords:** gut microbiome, host health, bioindicators, causal relationships, therapeutic strategies

Half a century ago, the tripartite interaction “disease is the outcome of imbalanced interplay among host, gut microbiome, and environmental variables” **[SIC]** was proposed (Snieszko, 1974). This paradigm has been supported by subsequent studies, which have revealed that the gut microbiota is a central hub that integrates environmental exposures with host genetic and immune signals, thereby determining host health outcomes (Kamada et al., 2013; Xiong et al., 2019). In particular, the transplantation of the gut microbiota from diseased individuals to healthy recipients induces the same disease and *vice versa*, revealing the causal role of dysbiosis in the gut microbiota and host health (Huang et al., 2020; Pandey et al., 2023). In this scenario, targeting the gut microbiota is a promising way to improve host health and treat disease. To achieve this, however, requires prior knowledge of the factors that affect the host gut microbiome. In addition, the “one pathogen, one disease” or “one virulence gene, one disease” paradigm is insufficient to validate the causal roles of polymicrobial pathogens in a disease. Accordingly, “ecological Koch's postulates” (one dysbiosis, one gut microbiota, one disease) are proposed to interpret these infectious diseases (Pascale et al., 2018; Xiong, 2018). It is apparent that the term “dysbiosis” is too simple and vague to explain complex disease states, thereby limiting the mechanistic understanding of etiology and the definition of causal agents. Therefore, unraveling the mechanisms behind dysbiosis is fundamental to better comprehending disease progression within a “pathobiome” concept and ultimately to guiding strategies for disease prevention.

Recently, ecological approaches have been applied to identify polymicrobial pathogens and their etiologies. For example, by integrating ecological features of primary colonizers, keystone taxa that drive gut networks from healthy to diseased cohorts, biomarkers of health status, as well as their encoding virulence gene, polymicrobial pathogens are inferred for shrimp white feces syndrome (Lu et al., 2020), coral white band disease (Gignoux-Wolfsohn et al., 2017), and human carcinogenesis (Cai et al., 2023). In addition, interkingdom phagotroph predator-prey interactions intimately kill the winning pathogens, thereby sustaining host health (Lu et al., 2022; Wu et al., 2022). A common observation is that disease symptoms lag far behind disease onset. Unfortunately, once disease symptoms appear, it is difficult to regain health in advanced disease stages, including shrimp and fish diseases (Xiong et al., 2017; Mougin and Joyce, 2023), and especially human cancers (Sung et al., 2021). There is evidence that disruption in the gut microbiota gradually worsens during disease progression, preceding host disease symptoms (Xiong et al., 2017; Shen et al., 2021; Cai et al., 2023). In fact, the gut microbiome is a key etiological element in the onset and progression of disease (Feng et al., 2015; Xiong et al., 2017). For these reasons, early detection of these adverse disorders in the gut microbiota is of paramount importance for predicting disease incidence in the host. The disease onset stage provides a better opportunity for disease biocontrol, such as antagonizing probiotics, synbiotics (Xiong, 2018; Goh et al., 2022), and even personalized treatments (Rodríguez-Fernández et al., 2022), although the designation of gut microbiota-based therapies is still a slow journey to primetime.

In light of the above-mentioned concerns, this Research Topic aimed to explore recent developments in this area with a focus on (1) exploring the underlying mechanisms governing the interrelationships between Environments-Pathogens-The gut microbiota and host disease from a molecular and ecological perspective; (2) identifying and validating causative relationships between polymicrobial pathogens and host disease progression; and (3) establishing approaches for diagnosing the incidence and/or outcome of disease, especially in the early (“subclinical”) stages, and biocontrol strategies for preventing host disease.

In one study, Liao et al. explore how host-gut microbiota interactions respond to decapod iridescent virus 1 (DIV1) infection using a lethal concentration 50 (LC_50_) assay. DIV1 infection causes dose-dependent mortality in shrimp (*Metapenaeus ensis*). In this study, the authenticity of the gut transcriptome is selectively validated by RT-qPCR, revealing that the expression patterns of the tested genes are comparable between mRNA sequencing and qPCR. Thus, RNA-Seq-assayed gene expression profiles are reliable for evaluating the effects of DIV1 infection on the shrimp transcriptome. DIV1 infection activates pathways involved in virus invasion, replication, and host antiviral infection, including lncRNAs. Specifically, shrimp fight DIV1 infection by potentiating the expression of the Wnt signaling pathway, the p53 signaling pathway, the C-type lectin receptor signaling pathway, and others. However, DIV1 infection up-regulates shrimp NF-κB inhibitor cactus-like and toll-interacting proteins through the proliferation of gut pathogenic *Vibrio* and *Photobacterium* genera, which suppress the toll-like receptor (TLR)-mediated immune response. Invertebrate shrimp cells recognize pathogen-associated molecular patterns on microbial pathogens through TLRs; thus, the inhibited TLRs create a favorable condition for immune escape and further DIV1 infection.

A second study by Su et al. uses publicly available datasets with large sample sizes of gut microbiota and gut microbial metabolites, thereby enabling them to obtain precise estimates and high statistical power. In this study, gut microbiota and gut microbial metabolites are deployed as exposure, while host health (here, low back pain, LBP) is used as the outcome. To correct for measured confounders, a multivariate Mendelian randomization analysis is conducted to obtain causal inferences between gut microbiota, gut microbial metabolites, and LBP outcome. As a result, 20 gut bacterial taxa and 2 gut microbial metabolites causally affecting LBP are examined. The results are consistent with the most available evidence. The workflow provides a causal effect of the gut microbiota-mediated mechanism of host health. Using similar procedures, Shi et al. identify causal relationships between gut bacterial taxa and five chronic respiratory diseases.

A review by Guevara-Ramírez et al. summarizes intrinsic and extrinsic factors that affect gut microbiota and hematologic cancer. Intrinsic variables, including host genetics, immunity, age, and health status, govern the gut microbiome. For example, host genetics can modify the expression of microbial receptors, determining the establishment of specific microbial species. Immune disorders disrupt microbial imbalances that contribute to carcinogenesis. Extrinsic variables, such as environmental exposures, diet, lifestyle, anticancer therapy, and stress, also affect the gut microbiota. Lymphoma is marked by an increased abundance of *Escherichia coli* and *Clostridium butyricum*, while leukemia is characterized by a decreased abundance of Lachnospiraceae and Ruminococcaceae. In contrast, myeloma is characterized by an enrichment of *Pseudomonas aeruginosa* and *Clostridium leptum* strains, with higher levels of *C*. *leptum* in the advanced stages of myeloma. *C*. *leptum* is a producer of butyrate that suppresses interleukin 17 (IL-17). Based on this knowledge, treatment with antibiotics or antibodies that block IL-17/IL-17R interactions delays myeloma progression. Thus, disease-specific strains may indicate different types and/or stages of hematologic disease. Moreover, the biology of disease-specific strains could guide the identification of therapeutic strategies.

Recently, there has been increasing evidence for the role of gut microbes in promoting host health and preventing host disease through biocontrol strategies. In support of this, Chen et al. investigate how different dietary compositions affect the gut microbiota and digestive enzyme activities of black soldier fly larvae. High-protein, high-fat, and high-starch diets significantly alter the gut microbiota, leading to subsequent changes in digestive enzyme activities. Among these diets, a high-oil diet stimulates the diversity of gut microbiota, which is associated with better growth and survival of black soldier fly larvae. By this logic, oil supplementation could improve the performance of black soldier flies in treating food waste for environmental protection. Geo et al. use Qishen Granule (QSG) to treat rats with heart failure. QSG significantly enriches gut Bacteroidetes and Prevotellaceae populations, thereby improving gut structure and barrier protection. In addition, QSG arranges mitochondria, alleviates swelling, and improves crest structural integrity. As a result, cardiac function and cardiomyocyte alignment are improved in heart failure rats. Therefore, QSG may potentiate cardiac function by regulating gut microecology, providing a viable therapeutic strategy for heart failure. Moreover, a review by Feng et al. summarizes the application of probiotics in the treatment of autism spectrum disorders (ASD) in humans from the perspective of a gut-brain axis. There are strong and positive associations between gut microbiota dysbiosis, gastrointestinal abnormalities, and ASD symptom severity. Dysbiosis in the gut microbiota causes immune system disorders and gut microbial metabolites. For example, elevated gut *Clostridia* species and *Pseudomonas stutzeri* strains produce high levels of *p*-cresol in individuals with ASD, leading to restricted social behavior and recognition. Conversely, suppressed gut *Lactobacillus reuteri* and *Bacteroides dentium* cause a reduction in aminobutyric acid, resulting in anxiety and depression-like behavior and stress responsiveness. Accordingly, supplementation with *B*. *longum* ameliorates microglia activity. *Lactobacillus* strains reverse valproic acid-induced apoptosis and degeneration in the cerebellum. Thus, probiotic supplementation improves ASD by regulating the gut–brain axis. Currently, an established probiotic protocol is lacking, resulting in a diversity of probiotic strains, concentrations, and treatment frequencies among studies. However, preclinical evidence has demonstrated recovery of brain function and improvement in ASD after probiotic supplementation.

## Perspectives

In conclusion, this Research Topic provides multidisciplinary knowledge on host-gut microbiota and gut microbial metabolites in response to disease and potential therapeutic strategies, such as a high-oil diet, QSG, and probiotics. Emphasis is placed on strategies targeting the gut microbiome to mitigate host disease. Nevertheless, the available data are cross-sectional, thus limiting the capacity to establish a cause-and-effect relationship between the gut microbiota and host health. Therefore, conducting longitudinal studies that integrate the microbiota across disease progression is essential to gaining a comprehensive understanding of this causal relationship. In addition, although the gut microbiome data have been deposited in public databases, the related covariates are descriptive or missing, making additional subgroup analyses necessary to rule out the confounders in an unbiased manner.

## Author contributions

JX: Conceptualization, Project administration, Writing—original draft, Writing—review & editing. ZS: Writing—original draft.
